# SARS-CoV-2 and HIV: Impact on Pulmonary Epithelial Cells

**DOI:** 10.3390/life12091317

**Published:** 2022-08-26

**Authors:** Nicholas J. Evans, Alina C. Schneider, Isabel Castro-Piedras, Ava G. Oliver, Alexandria B. Mabry, Amanda K. Garcia, Maria del C. Velez-Colon, Jacob Nichols, Matthew B. Grisham, Kevin Pruitt, Edu B. Suarez-Martinez, Sharilyn Almodovar

**Affiliations:** 1 Department of Immunology & Molecular Microbiology, School of Medicine, Texas Tech University Health Sciences Center, Lubbock, TX 79430, USA; 2 Department of Biology, University of Puerto Rico, Ponce, PR 00716, USA; 3 Department of Internal Medicine, School of Medicine, Texas Tech University Health Sciences Center, Lubbock, TX 79430, USA; 4 Department of Basic Sciences, Ponce Health Sciences University, Ponce, PR 00732, USA; 5 Center for Tropical Medicine and Infectious Diseases, School of Medicine, Texas Tech University Health Sciences Center, Lubbock, TX 79430, USA

**Keywords:** SARS-CoV-2, HIV, co-infection, polyparasitism, innate, adaptive, immune, air liquid interphase (ALI)

## Abstract

The SARS-CoV-2 pandemic provides a natural opportunity for the collision of coronavirus disease-2019 (COVID-19) with chronic infections, which place numerous individuals at high risk of severe COVID-19. Infection with Human Immunodeficiency Virus (HIV), a global epidemic, remains a major public health concern. Whether prior HIV+ status exacerbates COVID-19 warrants investigation. Herein, we characterized the impact of SARS-CoV-2 in human bronchial epithelial cells (HBECs) previously exposed to HIV. We optimized the air-liquid interface (ALI) cell culture technique to allow for challenges with HIV at the basolateral cell surface and SARS-CoV-2 spike protein on the apical surface, followed by genetic analyses for cellular stress/toxicity and innate/adaptive immune responses. Our results suggest that the IL-10 pathway was consistently activated in HBECs treated with spike, HIV, or a combination. Recombinant spike protein elicited COVID-19 cytokine storms while HIV activated different signaling pathways. HIV-treated HBECs could no longer activate NF-kB, pro-inflammatory TRAF-6 ubiquitination nor RIP1 signaling. Combinations of HIV and SARS-CoV-2 spike increased gene expression for activation of endoplasmic reticulum-phagosome pathway and downregulated non-canonical NF-kB pathways that are key in functional regulatory T cells and RNA Polymerase II transcription. Our in vitro studies suggest that prior HIV infection may not exacerbate COVID-19. Further in vivo studies are warranted to advance this field.

## 1. Introduction

Today and throughout history, our world has been challenged by many pandemics, from the black plague in Europe between 1346 and 1352 to the start of the worldwide spread of Human Immunodeficiency Virus (HIV) in the 1980s, to our most current pandemic caused by the outbreak of SARS-CoV-2 in 2019. Our life is in constant dynamic immune shifts in response to such challenges. At least 79 million people have been infected with HIV since the beginning of that pandemic, with 36 million lives claimed by AIDS according to the UNAIDS. More recently, the COVID-19 pandemic alone has affected ~595 million total cases worldwide and ~6.4 million deaths according to WHO (August 2022). It is important to take into consideration that the COVID-19 pandemic represents an additional pathogenic challenge to the global population and that individuals with pre-existing infections are at especially high risk for developing severe COVID-19 [1,2].

The infection with HIV comprises an older, well-established pandemic (or global epidemic) that remains an active and major public health concern. An estimated 38.4 million people are living with HIV (PLWH) globally [3]. Even with relentless efforts to optimize antiretroviral therapy (ART) over the last 25 years, HIV/AIDS remains a chronic disease. As such, HIV infection still requires close monitoring to detect drug-resistant variants, as well as non-compliance to therapy, because both can fuel disease progression and HIV transmission. PLWH were already susceptible to a myriad of comorbidities before being hit by the COVID-19 pandemic [4,5,6,7]. The collision of these two pandemics provides a unique opportunity to study the SARS-CoV-2 interaction with pre-existing pathogens such as HIV.

The early use of ART in PLWH has been associated with better COVID-19 prognosis, albeit with a limited sample size [8]. Based on several other clinical studies, it remains unclear how exactly SARS-CoV-2 infection impacts immunosuppressed patients, but there seems to be no tendency for worse outcomes [9,10,11,12,13]. ART has in fact also been proposed as a potential for the treatment of COVID-19 in the general population [8,14]. There are also clinical studies stating that a previous HIV infection leads to a worse progression of COVID-19, increased mortality rate [15,16], is more likely to develop cardiovascular disease [15,17], and has increased comorbidity of HIV with diabetes mellitus and hypertension [18]. While others state there are similar mortality rates but differing chronic disease profiles and more severe complications [19,20]. There are apparent incongruences in the current scientific literature regarding clinical observations in PLWH with COVID-19. To this end, rigorous basic research aimed at increasing the understanding of molecular mechanisms at play between SARS-CoV-2 and HIV and its impact on the host is critical. Nevertheless, the pathogenicity of HIV as a bloodborne pathogen and SARS-CoV-2 as an airborne pathogen that further gains access into the bloodstream complicates scientific methodology, particularly in academic research settings without access to BSL-3 facilities.

Herein, our study aimed at developing a double pathogen in vitro model for the characterization of the interactions between HIV and SARS-CoV-2 in the human pulmonary epithelium. Previous studies have confirmed productive HIV infection in epithelial cells using the air-liquid interface (ALI) cell culture technique [21,22]. In this novel study, we optimized the ALI technique to allow for appropriate polarization of human bronchial epithelial cells (HBECs) followed by challenges with infectious HIV on the basolateral cell surface and SARS-CoV-2 protein on the apical surface without compromising the expression of its key receptor Angiotensin-Converting Enzyme 2 (ACE2). Furthermore, we investigated the impact of the virus challenges by measuring gene expression changes in the pulmonary epithelium.

## 2. Materials and Methods

**Figure 1:**
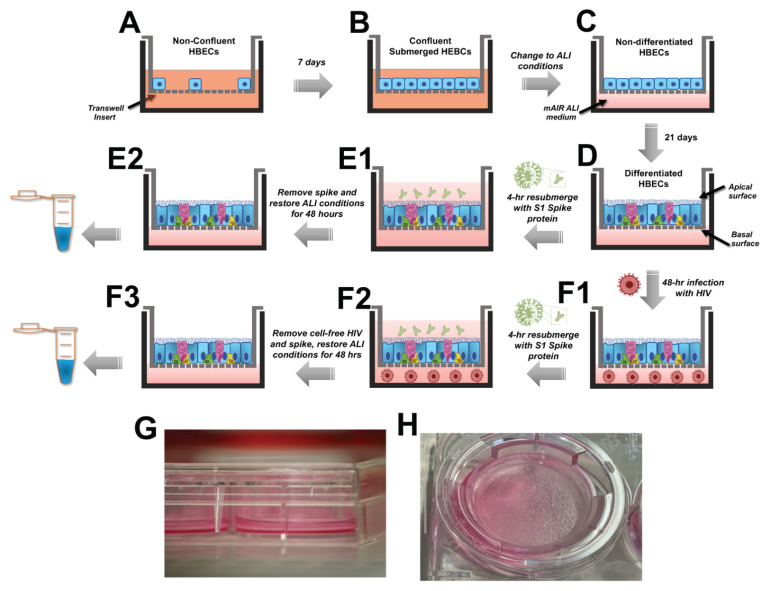
Schematic representation of the ALI System and process for co-challenge with HIV and SARS-CoV-2 Spike protein. The steps to culture HBE4 cells under ALI conditions are shown. (**A**) depicts HBE4 cells cultured on 0.4 um membrane inserts (transwells) using supplemented keratinocyte medium and allowed to reach confluency after 7 days as depicted in (**B**). (**C**) shows confluent HBE4 cells changed to ALI conditions by replacing the supplemented keratinocyte medium by fresh mAir media mixture applied only to the bottom chamber of the transwell. (**D**) shows differentiated HBE4 cells after 21 days under ALI conditions, with media changes every 48 h. At this point, cells were either treated with spike protein alone, or HIV, or their combination as follows. (**E1**) shows differentiated HBE4 cells resubmerged for exposure to SARS-CoV-2 spike protein (or vehicle) on the apical surface for 4 h. (**E2**) shows cells under restored ALI conditions (spike protein removed) for 48 h prior to processing for molecular studies. (**F1**) shows differentiated HBE4 cells exposed to cell-free HIV on the basolateral surface for 44 h. (**F2**) shows HIV-treated cells resubmerged for exposure to SARS-CoV-2 spike protein (or vehicle) on the apical surface for 4 h, for a total co-exposure time of 48 h. (**F3**) shows co-challenged cells under restored ALI conditions (HIV and spike protein removed) for an additional 48 h prior to processing for molecular studies. (**G**) shows a photograph of the transwell system and (**H**) shows cells with visible mucus production. Abbreviations: ALI, air-liquid interface, HBEC, human bronchial epithelial cells.

**Culture of human pulmonary epithelial cells.** We used human bronchial epithelial cells HBE4-E6/E7-C1 (ATCC #CRL-2079) in this study, grown in Gibco Keratinocyte Serum-Free (KSF) Media (Gibco, Paisley, Scotland cat# 17005042), supplemented with human recombinant epidermal growth factor and bovine pituitary extract, as per manufacturer protocol. According to the vendor, HBE4 cells were derived from the normal bronchial epithelium of a 60-year-old man undergoing a lobectomy due to poorly differentiated adenocarcinoma and immortalized using the HPV 16 E6/E7 system. For the purposes of this study, cells were grown on transwell polyester or polycarbonate membrane cell culture inserts in 6-well plates (24 mm diameter, 0.4 um pore, polyester: Corning Carlsbad, California, USA, cat# 3450; polycarbonate: Corning, cat# 3412) or 100 mm dishes (75 mm diameter, 0.4 um pore, polycarbonate, Corning cat# 7910). Cells in 6-well transwell plates were seeded with 200,000 cells suspended in 1 mL of complete KSF medium on the top chamber and 2 mL of medium added to the bottom chamber (Figure 1G). For 75 mm transwells, 1.2 million suspended in 5 mL of KSF medium were seeded on the top of the transwell and 9 mL of medium was added to the bottom (Figure 1A). All cells were incubated at 37 °C with 5% CO_2_. Media were changed every 2–3 days during cell culturing. Cells became confluent in 3–5 days and were allowed to form tight junctions for 5–10 additional days before proceeding with the Air-Liquid Interface (ALI) cell culture technique (Figure 1B).

**ALI Cell Culture.** For ALI, the KSF medium was removed and replaced with mAIR medium, as previously optimized by Luengen et al. [23]. Briefly, mAIR consists of a mix of the Airway Epithelial Cell Growth Medium Kit (AECGM, PromoCell, Heidelberg, Germany, #C-21160), excluding the supplement triodo-L-thyronine, and adding 50 mM retinoic acid, along with bovine pituitary extract, epidermal growth factor, insulin, hydrocortisone, epinephrine, and transferrin as supplemented in the kit. This was then mixed 1:1 with Dulbecco’s Modified Eagle Medium (DMEM, Gibco #11995-065) previously supplemented with 50 mL of fetal bovine serum (Corning #35-011) and 5 mL of Penicillin-Streptomycin (Sigma-Aldrich, Saint Louis, MO, USA #P4333). The resulting mAIR media was then added to the bottom chamber of the transwell (2 mL in each 6-well plate transwell, or 9 mL in 75 mm transwell), as depicted in Figure 1C. The formation of tight junctions is crucial in this process; the medium in the bottom chamber (basal side) should not compromise the integrity of the apical cell surface (air). To this end, defects in the preservation of ALI conditions over the culturing period are usually evident by leaky transwells. In addition, effective cell differentiation was indicated by visible mucus production, as shown in Figure 1H, coupled with qPCR confirmation of gene expression for cilia, goblet cells, neuroendocrine, and Club cells. Expression of ACE-2 receptor was confirmed by qPCR and Western blotting. We used cells after 21 days in ALI conditions (Figure 1D); leaky transwells were excluded from the study.

**Challenge of epithelial cells with SARS-CoV-2 spike protein and HIV.** HBE4 cells were exposed to SARS-CoV-2 recombinant spike protein (Wuhan-Hu-1, accession QHD43416, sourced from RayBiotech #230-30162, Peachtree Corners, GA, USA). The biological activity of this spike protein was confirmed by binding to the human ACE-2 receptor by ELISA (Human ACE-2 ELISA Kit, RayBiotech #ELH-ACE2-1). The spike protein was added to the apical surface of HBE4 cells (top chamber) at either 50 ng/mL or 5 μg/mL concentration and incubated for 4 h (Figure 1(E1)). Cell culture medium was replaced with fresh medium and cells were allowed to recover for 48 h prior to processing for molecular endpoints (Figure 1(E2)). For treatments with HIV, infectious cell-free HIV (HIV-1_NL-D,_ kindly provided by Dr. Tsunetsugu-Yokota, National Institute of Infectious Diseases, Tokyo, Japan [24,25,26]) was added to the bottom chamber of the transwells and incubated for 48 h (Figure 1(F1)). Independent cell cultures were exposed to both pathogens by first treating with HIV for 48 h (basolaterally), and then with spike protein (apically) for the last 4 h of the 48-h treatment (Figure 1(F2)). Cell culture media were replaced with fresh media and cells were allowed to recover for an additional 48 h prior to processing for RNA or protein extractions (Figure 1(F3)).

**RNA Extraction and PCR arrays.** Total RNA extractions were performed using the Aurum Total RNA Mini Kit (Bio-Rad, Hercules, CA, USA #732-6820), followed by concentration measurements using a NanoDrop spectrophotometer, cDNA synthesis using RT^2^ First Strand Kit (Qiagen, Germantown, MD, USA), and quality control check using RT^2^ RNA QC PCR arrays (Qiagen #PAHS-99) coupled with SYBR Green qPCR Mastermix (Qiagen #330529). We used qPCR with primers specific for differentiation markers of ciliation, Club cells (non-ciliated epithelial cells), pulmonary neuroendocrine cells, and Goblet cells as listed in Table 1. We utilized RT^2^ Profiler PCR Arrays for screening of regulatory genes, specifically the Human Stress and Toxicity Pathway Finder (Qiagen #PAHS-003) and the Human Innate and Adaptive Immune Responses (Qiagen #PAHS-052) as per manufacturer protocols. Gene arrays were performed in Bio-Rad CFX96 real-time PCR system. The C_T_ values were uploaded onto the Qiagen data analysis web portal (http://www.qiagen.com/geneglobe, accessed on 28 January 2021) for calculation of fold change/regulation using the delta delta C_T_ method, followed by (delta C_T_ (Test Group)—delta C_T_ (Control Group). Fold changes were calculated using 2^–∆∆Ct^) formula, using a C_T_ cutoff set to 35. Data normalization was done using the geometric means of all three housekeeping genes β2-microglobulin (B2M), glyceraldehyde-3-phosphate dehydrogenase (GAPDH), and Ribosomal protein lateral stalk subunit P0 (RPLP0).

**Western Blot.** Whole cell protein extracts were generated by using either RIPA buffer (Cell Biolabs #AKR-190, San Diego, CA, USA) or Pierce IP Lysis buffer (Thermo Scientific, Rockford, IL, USA #87788) supplemented with cOmplete^TM^ Protease Inhibitor Cocktail (Sigma Aldrich, Mannheim, Germany, #11697498001). Cell lysates were rocked in the cold room for 30 min, followed by centrifugation at 12,000× *g* for 10 min at 4 °C. Cleared supernatants were collected for measurement of protein concentration using the colorimetric DC Protein Assay (Bio-Rad, Hercules, CA, USA #5000111) on an Eppendorf BioPhotometer. Lysates were then aliquoted and stored at −80 °C to prevent degradation. Proteins (50 μg total) were mixed with 6x sample loading buffer and boiled for 5 min prior to separation by electrophoresis on either 10% TGX gels (Bio-Rad #456-1034) or 12% TGX 10-well Fast Cast Acrylamide gels (Bio-Rad #1610175). Proteins were transferred to Immuno-Blot PVDF membranes (Bio-Rad #162-0177) using the wet transfer method. Membranes were blocked in 5% TBS-milk at 4 °C overnight, followed by incubations with primary antibodies at 4 °C overnight and incubation with secondary antibodies for 1 h at room temperature. The antibodies used in this study are listed in Table 2. We washed the membranes with TBS-0.01% Tween (3 washes, 5 min each) in between incubations and prior to imaging. Blots were visualized after incubating the membranes with Radiance Plus chemiluminescent substrate (Azure Biosystems, Dublin, CA, USA) for 5 min with rocking; images were documented using an Azure c300 chemiluminescent Western blot imaging system and densitometry analyses were performed using FIJI [27].

Statistical analyses. Data are provided as means ± SEMs. Statistical significance was determined using the 2-tailed Student *t*-test, ANOVA and Kruskal–Wallis test, as appropriate with corrections for multiple comparisons. All the qPCR data were analyzed using Qiagen data analysis web portal (http://www.qiagen.com/geneglobe, accessed on 17 December 2021) with a Fold-Change represented as (2^–∆∆Ct^) normalized to gene expression (2^–∆∆Ct^), and *p*-values calculated based on a Student’s t-test of the replicate 2^–∆∆Ct^. Western Blot datasets were analyzed using FIJI. Statistical analysis and graph design were performed using GraphPad software for Mac (Prism version 9.3.1, San Diego, CA, USA).

## 3. Results

### 3.1. Validation of HBECs Culturing Conditions with SARS-CoV-2 Spike Protein In Vitro

To accomplish the goal of this study, we first confirmed that the cell culture conditions of the HBE4 epithelial cells were appropriate for responses to the viral pathogens. Most of the published studies with HBE4 cells cultured under air-liquid interface (ALI) conditions undergo cell polarization and can differentiate into several specialized cell types known to populate the airways.

**Figure 2:**
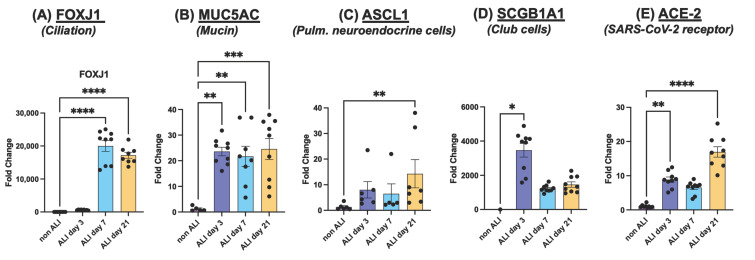
**Characterization of gene expression in HBE4 cells cultured in ALI conditions.** Cells were cultured using standard cell culture techniques (submerged in medium, non-ALI) or in ALI. Cells were collected at the indicated time points and processed for RNA extractions followed by quantitative PCR as described in the Methods section. We measure gene expression for several stages in the differentiation process including ciliation (**A**), production of mucin (**B**), pulmonary neuroendocrine cells (**C**), Club cells (**D**), as well as expression of ACE-2 receptor (**E**). Fold changes were calculated using 2^–∆∆Ct^) formula, using a C_T_ cutoff set to 35. Data normalization was done using the geometric means of all three housekeeping genes β2-microglobulin (B2M), glyceraldehyde-3-phosphate dehydrogenase (GAPDH), and Ribosomal protein lateral stalk subunit P0 (RPLP0). Data points are represented by black circles. Statistical differences were calculated using the “Kruskal–Wallis test Anova results” in GraphPad, *p* < 0.05 symbolized by *, *p* < 0.01 symbolized by **, *p* < 0.001 symbolized as *** and *p* < 0.0001 symbolized as ****.

First, we confirmed the stages of cell differentiation in the ALI culture parameters by measuring gene expression associated with specific hallmarks of mature ALI cultures, compared to cells cultured using the standard cell culture technique. Specifically, Forkhead Box J1 (FOXJ1), a marker of ciliation, showed a significant increase in gene expression after day 7 of ALI, (Figure 2A). In addition, HBE4 cells are known precursors of mucus-producing Goblet cells, which can be surveyed by expression of Mucin 5AC (MUC5AC). We found significantly increased gene expression of MUC5AC as early as day 3 of ALI and sustained over the 21-day period (Figure 2B). As basal HBE4 cells differentiate, bronchiolar exocrine cells (also known as Club or Clara cells) can also develop along with pulmonary neuroendocrine (NE) cells, albeit at low frequencies [28,29]. We used the Secretoglobin family 1A member-1 (SCGB1A1) and Achaete-Scute Family bHLH Transcription Factor 1 (ASCL1) as genetic markers for Club and NE cells, respectively. Our results show significantly increased gene expression for both markers starting at day 3 of ALI conditions (Figure 2C,D). With these data, we decided to let the HBECs differentiate for 21 days for optimal results.

**Figure 3:**
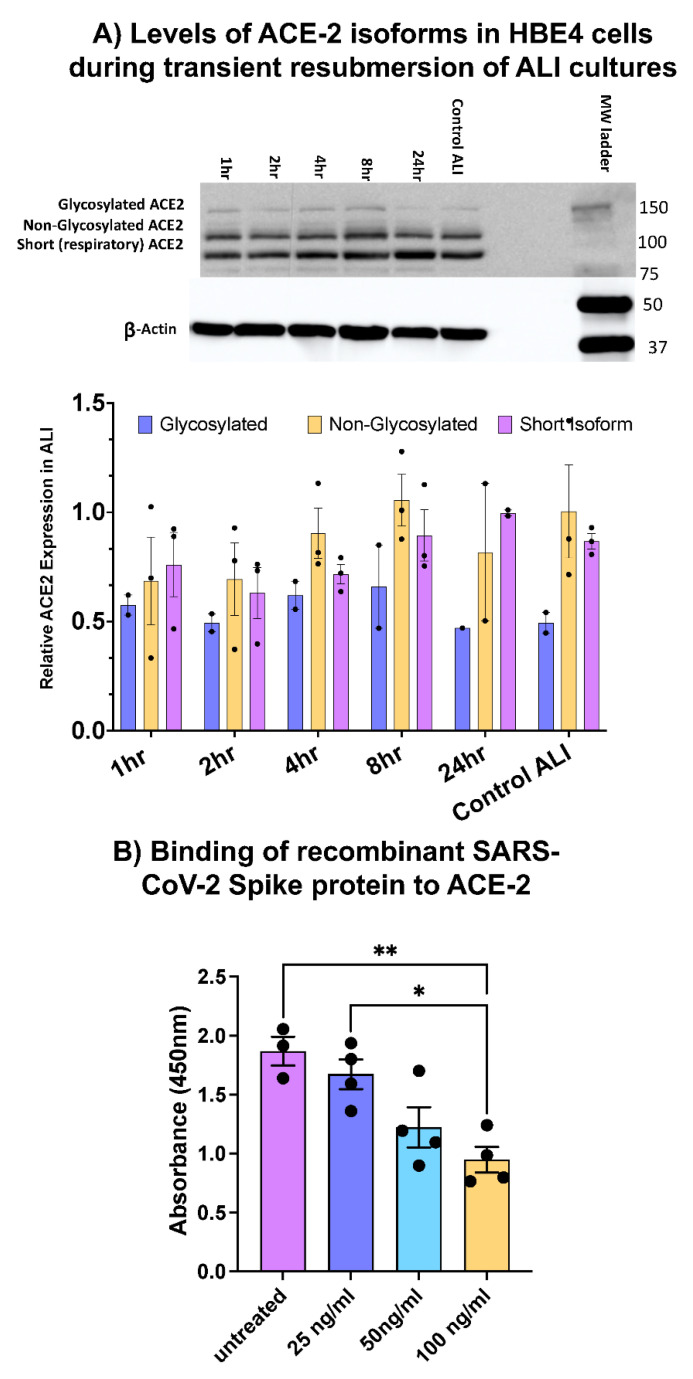
**Functionality of recombinant SARS-CoV-2 spike protein and levels of ACE2 expression in ALI cultures.** (**A**) shows the levels of expression of ACE2 isoforms (glycosylated, non-glycosylated, and short), as detected by Western blot, in 21-day ALI cultures that were resubmerged for the indicated times. Data are shown as ACE2 expression normalized to β-actin, SEM. Statistical differences were assessed using Kruskal–Wallis tests for multiple comparisons. (**B**) shows the results of recombinant SARS-CoV-2 binding to ACE-2 assays done by ELISA, as described in the Methods section. Data are indicated as mean absorbance at 450 nm, SEM; each datapoint is represented with black circles. Statistical differences were calculated using Student’s *t*-Test in GraphPad Prism, *p* < 0.05 symbolized by * and *p* < 0.01 symbolized by **.

Given the nature of SARS-CoV-2 as a respiratory pathogen, the expression of ACE2 as an essential receptor for SARS-CoV-2 interactions with the airway epithelium [30] was considered. The ACE2 receptor is known to exist in three distinct isoforms in the pulmonary epithelium; a functional form (120 kD) with varying degrees of glycosylations as described by Shajahan et al. [31], a non-glycosylated form (100 kD) [32], and a novel non-glycosylated shorter isoform of 75 kD that lacks the binding sites for SARS-CoV-2 spike protein, as recently discovered by Blume et al. [33]. We confirmed increased ACE2 genetic expression in HBE4 cells under ALI conditions by qPCR, with the most noticeable increase at day 21 of ALI (Figure 2E). While Jia et al. observed that ACE2 increases with ALI, they also observed downregulation of ACE2 upon resubmersion of cells in the ALI culture [30]. This is a crucial consideration given that the recombinant SARS-CoV-2 S1 spike protein we used in these studies is provided in liquid solution. Therefore, any treatments with spike protein would require resubmersion of HBE4 ALI cell cultures for the duration of the experiment, with potential implications in cell de-differentiation including decreased susceptibility to S1 effects [30]. To clarify this, we determined the window of time that the ALI culture can be resubmerged before noticeable changes in the expression of ACE2 isoforms, compared to an ALI control that was never resubmerged. Considering that exposures to SARS-CoV-2 are transient, ALI cultures were resubmerged for 1, 2, 4, 8, and 24 h before immediate cell lysis for protein extractions, followed by Western blot analyses. Our results show varying levels of all three isoforms of ACE2 (Figure 3A), normalized to β-Actin. Interestingly, ALI controls that were never resubmerged trended to show lower expression of glycosylated ACE2. To ensure consistency in treatment conditions, we designated 4 h as the appropriate incubation time with spike, which would not compromise the expression of ACE2 receptors in transiently resubmerged cells.

### 3.2. SARS-CoV-2 Spike Protein Alters Genes Associated with Innate and Adaptive Immunity as Stress Response

In an effort to investigate how HBECs respond to the challenge of SARS-CoV-2, we treated cells with either 50 ng/mL or 5 ug/mL S1 recombinant protein. First, we confirmed the biological activity of spike protein by its ability to bind to ACE-2 receptor, using ELISA pre-coated with anti-human ACE-2 antibody. Briefly, three different concentrations of spike protein were tested (at 25, 50, and 100 ng/mL), compared to untreated control. Any exogenous (and active) spike protein would bind to the ACE-2 receptor and block the binding site for anti-ACE-2 antibodies added post-spike treatments. Figure 3B shows a consistent decrease in ACE-2 absorbance with increasing concentrations of spike protein, which confirmed the biological activity of this recombinant protein prior to use in cell culture. Second, we initially surveyed changes in RNA expression of 84 genes related to several categories including oxidative stress, hypoxia, osmotic stress, cell death, inflammatory response, DNA damage and response, and unfolded protein response, using the Human Stress and Toxicity Pathway Finder PCR array. The C_T_ results were uploaded into the Qiagen GeneGlobe Data Analysis Center and normalized using RPLP0, GAPDH, and B2M housekeeping genes. We considered fold-regulation changes of >1.5 and the top hits with significant *p*-values are summarized in Table 3. Notably, the top hits that resulted from spike treatments are within the inflammatory response gene category, with significant downregulation of C-C Motif Chemokine Ligand-2 (CCL2), and significant upregulation of interleukin-1 alpha (IL-1α) and interleukin-1 beta (IL-β).

We further investigated the HBECs genetic responses to the SARS-CoV-2 spike using the Qiagen RT^2^ Profiler Human Innate and Adaptive Immune Response Array. Treatments of HBEC with 50 ng/mL of recombinant spike protein for 4 h followed by 48-h recovery resulted in >4-fold and statistically significant upregulation of Interferon-induced MX dynamin-like GTPase 1 (MX1), and C-X-C motif chemokine ligand-10 (CXCL10). In addition, we measured >2-fold significant upregulation of C-C motif chemokine ligand-5 (CCL5), IL-1β, IL-1α, and DExD/H-box helicase 58 (DDX58), as shown in Table 4.

### 3.3. Stress Responses of HIV-Infected Epithelial Cells to SARS-CoV-2 Spike Protein

Once we established the scope of the changes in gene expression of HBE4 cells treated with SARS-CoV-2 spike protein, we analyzed how SARS-CoV-2 spike protein affects gene expression in HBECs already compromised with HIV. To this end, we co-challenged the HBE4 cells with HIV and spike protein, as described in Figure 1F. Specifically, we treated cells for 48 h with HIV added to the basolateral portion of the transwell (bottom chamber), and recombinant spike protein was added to the apical side for 4 h (*n* = 6). Additional experimental groups included HBE4 cells treated with HIV (*n* = 4) or spike only (*n* = 5), as well as controls treated with vehicle (*n* = 8). Cells were harvested for RNA extractions and processed for gene profiling using the Human Innate and Adaptive Immune Response targeted PCR array.

**Figure 4:**
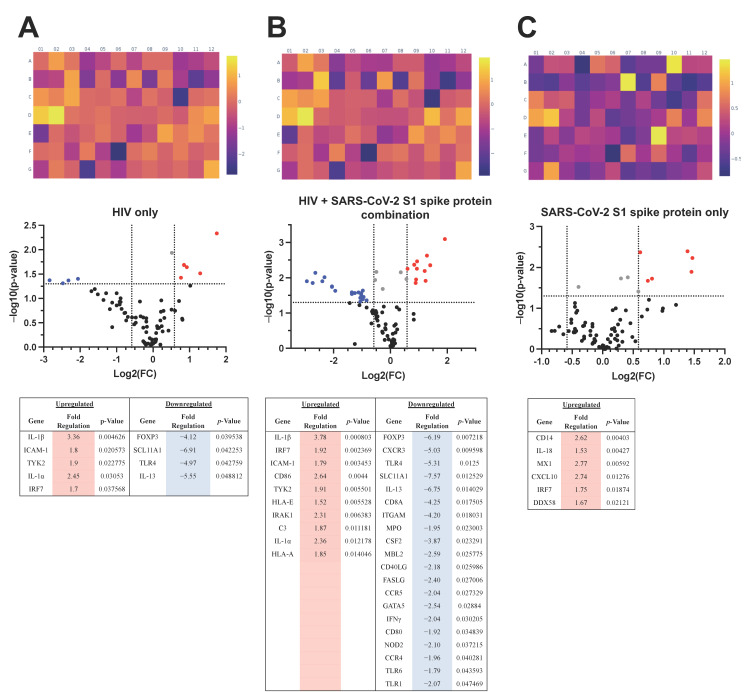
**Changes in RNA Expression in HBECs treated with SARS-CoV-2 S1 protein, HIV, and HIV S1.** (**A**) summarizes the results for HBECs treated with HIV only; (**B**) for co-challenge with infectious HIV and SARS-CoV-2 S1 spike protein; and (**C**) for HBECs treated with SARS-CoV-2 S1 spike protein only. The heat map depicts the log2 of the fold changes. The Volcano Plot identifies significant gene expression changes by plotting the log2 of the fold changes in gene expression on the x-axis versus their statistical significance on the y-axis. The two outer vertical lines indicate the selected fold regulation threshold. The horizontal line indicates the selected *p*-value threshold. Genes with data points in the far upper left (down-regulated) and far upper right (up-regulated) sections meet the selected fold regulation and *p*-value thresholds. Red dots symbolize significantly upregulated and blue dots symbolize significantly downregulated genes; the gray dots represent genes that did not meet the fold change criteria and the black dots represent unchanged genes in the panel. The tables below list the significant fold regulations for each version of the HBEC challenge. Sample size: untreated control *n* = 8; S1 protein only *n* = 5; HIV only *n* = 4; HIV + S1 protein *n* = 6.

In the presence of HIV only, HBECs exhibited significant >2-fold upregulation of IL-1α and IL-1β, as well as significant >4-fold downregulation of Forkhead Box P3 (FOXP3), Toll-like receptor-4 (TLR4), interleukin-13 (IL-13), and Solute Carrier Family 11 Member 1 (SLC11A1), as shown in Figure 4A. Interestingly, the combination of HIV and SARS-CoV-2 spike protein promoted >2-fold significant upregulation of Interleukin 1 Receptor Associated Kinase-1 (IRAK1), IL-1α, CD86, and IL-1β. In addition, we observed >2-fold downregulation of Toll-like receptor-1 (TLR1), interferon g (IFNγ), Nucleotide Binding Oligomerization Domain Containing-2 (NOD2), C-C motif chemokine receptor-5 (CCR5), CD40 ligand (CD40LG), GATA Binding Protein-5 (GATA5), Fas ligand (FASLG), mannose-binding lectin-2 (MBL2), as well as >4-fold significant downregulation of Integrin Subunit Alpha M (ITGAM), CD8a, C-X-C motif receptor-3 (CXCR3), TLR4, FOXP3, IL-13, and SLC11A1. Data of genetic changes associated with co-challenge of HBECs with HIV and spike protein are summarized in Figure 4B. In HBE4 cells treated with spike protein, we confirmed significant upregulation of CXCL10 and MX1, as well as CD14, compared to controls (Figure 4C). 

Next, we sought to understand the biological significance of our gene expression findings with Reactome pathway analyses. Our results show that treatments of HBECs with SARS-CoV-2 spike protein led to significant upregulation of IL-1 family signaling, as well as pro-inflammatory interferon producing IL-18 signaling pathways and first-line antiviral defense through increased expression of interferon-regulatory factors (IRF). None of the signaling pathways targeted by the innate and adaptive immune response PCR array were significantly downregulated in cells treated with spike protein.

When challenged with HIV as a sole pathogen, HBECs pathway analyses suggest significant activations in NK-cell receptor Dectin and pyroptosis (a pro-inflammatory form of cell death induced by infections). In contrast to spike, pathway analyses of HIV-treated HBECs suggested significant downregulation of several pathways, including apoptosis mediated by Toll/IL-1R TIR-domain-containing adapter-inducing IFN-beta (TRIF), IRAK-2 mediated activation of Transforming growth factor-β (TGF-β)-activated kinase 1 (TAK1) complex, and Receptor-interacting protein 1 (RIP1) signaling. Opposite to the spike, HIV treatments significantly downregulated IL-18 signaling and IRF pathways.

According to Reactome over-representation pathway analyses, co-challenges of HBECs with HIV and SARS-CoV-2 spike protein resulted in significant activation of endoplasmic reticulum-phagosome pathways. In addition, HIV + spike combinations on HBECs led to significant downregulation of Toll-like receptor signaling, FOXO-mediated transcription of cell death genes, and TNFR2 non-canonical NF-kB pathway.

**Figure 5:**
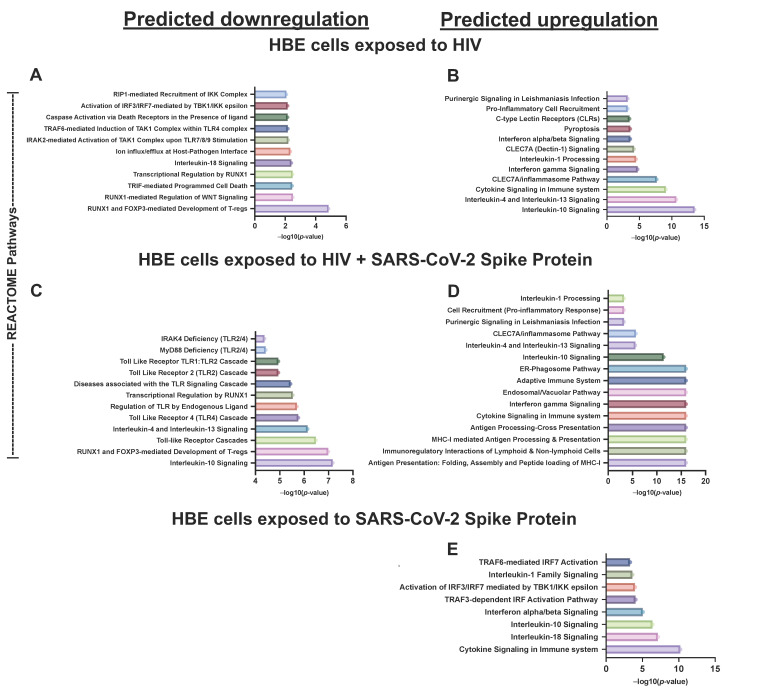
**Predicted pathways that are significantly influenced by SARS-CoV-2 S1 protein, HIV and HIV + S1 treated HBECs:** The bar graphs show Reactome pathways found to be significantly affected by the corresponding treatment. The x-axis shows the significance as the −log10 of the *p*-value. (**A**,**B**) visualize the pathways affected by changes in gene expression in HBECs challenged with HIV, (**C**,**D**) for HBECs challenged with HIV and SARS-CoV-2, and (**E**) HBECs challenged with SARS-CoV-2 S1 protein. All pathways and their *p*-values were found at https://reactome.org (accessed on 21 August 2022).

**Figure 6:**
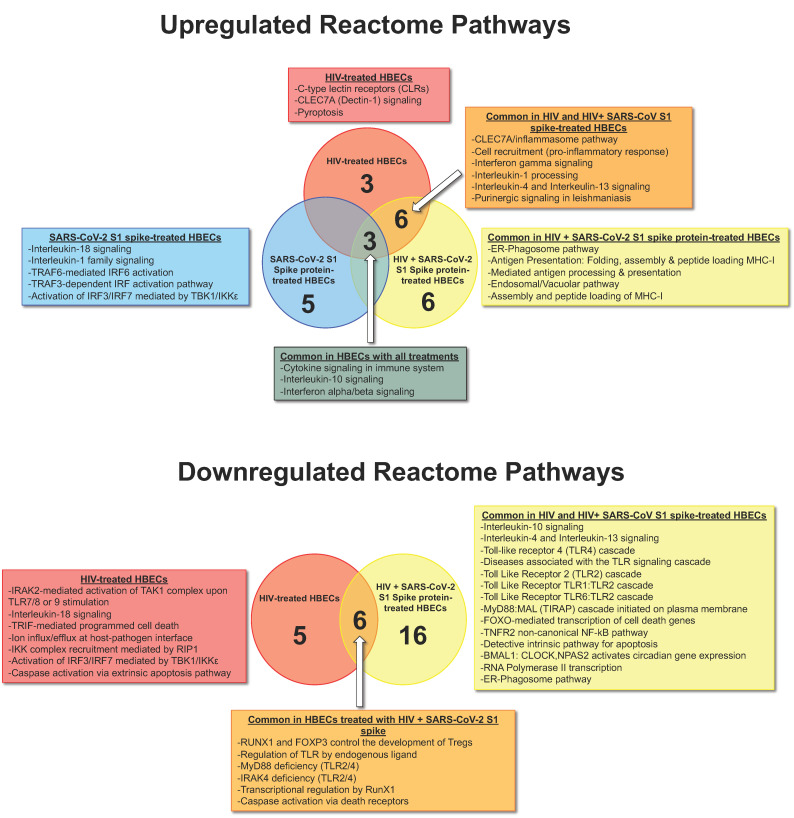
**Enrichment pathways analyses in HBECs treated with HIV, SARS-CoV-2 S1 spike protein, or combination.** We used the gene lists obtained from our gene expression analyses to perform over-representation pathway analyses using the Reactome pathway database (available at https://reactome.org/ (accessed on 21 August 2022)). The enriched pathways shown in Venn diagrams in the top panel indicate the upregulated pathways; the bottom panel shows downregulated signaling pathways. The blue shapes represent data from HBECs exposed to SARS-CoV-2 S1 Spike protein only; the red shapes represent data from HBECs treated with infectious HIV only, and the yellow shapes represent data from HBECs treated with the combination. The blended colors (orange and dark gray) represent signaling pathways that are common between the indicated treatments.

In the context of pathways common between HIV-treated HBECs and HIV + spike-treated cells, Reactome analyses identified several common pathways that were significantly activated including IL-4 and 13 signaling, as well as non-canonical inflammasome pathways known to be activated independently from caspase 8 but triggered by C- C-Type Lectin Domain Containing 7A (CLEC7A). We also found that treatments of HIV alone or HIV/spike combinations led to significant downregulation of signaling leading to the formation of T-regs, as well as caspase activation by death receptors. In all treatment types (S1, HIV, and combination), the IL-10 pathway was significantly upregulated (Figure 5). The signature signaling pathways that were enriched upon individual treatments or their combinations are summarized in Figure 6.

## 4. Discussion

Essentially everyone is susceptible to SARS-CoV-2 infection; however, individual implications of COVID-19 remain a mystery, particularly in high-risk individuals. With over half a billion cases of SARS-CoV-2 infections worldwide [34], the probability that a high number of PLWH experience co-infection with SARS-CoV-2 sometime in their lifetime is alarmingly high. Unfortunately, the prevalence and pathophysiological implications of the SARS-CoV-2/HIV polyparasitism remain to be clarified in this novel COVID era.

One of the curbs in many research laboratories willing to pursue timely COVID-19 research is the lack of biosafety level-3 facilities required to grow SARS-CoV-2 and hence, the incapability of pursuing research with live virus. The good news is that several surrogate tools including highly purified recombinant proteins, attenuated viruses, and pseudotyped virions have been readily available for COVID-19 research. The unsettling news is that the virus continues evolving leading to novel variants and its newest lineages might not be represented adequately in most of the biotechnology research tools to study it. In addition, the wide use of cell lines like A549 lung cells and HEK293 kidney cells might introduce variables inherent to the cancerous nature of such cell lines, depending on the experimental question. In addition, culturing under anatomically and physiologically relevant conditions is key to gaining reliable insights when investigating the impact of polyparasitism in COVID. For pulmonary airway epithelial cells, exposing the cells to air and liquid is key to cell polarization and the expression of relevant cell surface markers. For instance, a study by Jia et al. [30] established the importance of epithelial cell differentiation stages in mechanistic studies on interactions of beta-coronaviruses and ACE2 receptors. They demonstrated effective polarization of epithelial cells cultured in ALI conditions and its importance on ACE2 expression and susceptibility to viral infection. Moreover, they showed complete loss of ACE2 expression on epithelial cells resubmerged for 7 days after being in ALI conditions.

Our study was rigorously designed so that airway epithelial cells were co-challenged with HIV on the basal surface and SARS-CoV-2 on the apical surface, using spike protein as a proxy. Therefore, it was important for us to document the relevant markers of cell polarization and expression of ACE-2 receptors that are required in SARS-CoV-2 studies. Here, we show that SARS-CoV-2 spike protein added to human bronchial epithelial cells in culture was sufficient to significantly trigger interferon-producing anti-viral pro-inflammatory pathways associated with innate and adaptive immune responses. We found that the most profound changes were in MX-1 and CXCL10 expression, which are key in COVID-19. No significant downregulations in signaling pathways were measured by the PCR arrays. When challenged with HIV only or HIV/Spike combinations, we found dysregulation in a whole different gene set.

When challenged with HIV as a sole pathogen, HBECs responded by activating C-type lectin receptor signaling, which is essential in antifungal and antiviral innate immunity [35,36]. We also observed the activation pyroptosis pathways, which are one of the drivers of CD4 T-cell depletion in HIV-infected people [37]. In contrast to spike-treated cells, HIV-treated HBECs suggested significant downregulation of several pathways, including necroptosis and TAK1 complex. This implies that, in the context of HIV infection, HBECs could no longer activate NF-kB and could no longer stimulate TRAF-6 ubiquitination [38], nor the RIP1 signaling that activates pro-inflammatory cytokines via induction of NFkB pathways [39]. Unlike spike protein, HIV treatments significantly downregulated IL-18 signaling and IRF pathways. This is consistent with the known ability of HIV to evade IRF mainly through lysosomal degradation or caspase-dependent cleavage mediated by the Vpu accessory protein in HIV [40,41]. Notably, our novel method exposed HBECs to HIV for 48 h and to SARS-CoV-2 spike protein for 4 h, followed by another 48-h recovery. We acknowledge that a 48-h exposure to HIV may not translate to chronic HIV infection however, the relatively short exposure of HBECs to HIV was sufficient to induce significant changes in genetic expression portraying the well-known immune suppression induced by HIV. Moreover, most PLWH with access to ART achieve viral suppression after a period of active viral replication during the acute infection. With the exception of occasional blips in viral load, there is no prolonged cell exposure to infectious HIV in treated patients. For this reason, we believe that the model we propose here is still relevant to acute, untreated HIV infection. We also believe that this model can be easily adapted to include ART and extend the duration of the cultures to translate to chronic HIV infection.

Our results show that pulmonary epithelial cells previously compromised by HIV and then exposed to SARS-CoV-2 spike protein respond by activating the endoplasmic reticulum-phagosome pathway that is key in the cross-presentation of exogenous antigens to MHC class I molecules to CD8 cells [42]. HIV + spike combinations on HBECs also led to significant downregulation of Toll-like receptor signaling, FOXO-mediated transcription of cell death genes, TNFR2 non-canonical NF-kB pathway, which are key in the function of regulatory T cells, and RNA Polymerase II transcription pathways.

Common pathways activated by HIV and HIV + spike-treated cells included IL-4 and 13 signaling, as well as non-canonical inflammasome pathways known to be activated independently from caspase 8. Such pathways are independent of pathogen internalization and enable the host to harbor robust TH17 responses [43]. We also found that HIV alone or HIV/spike combinations significantly downregulated the forkhead box transcription factor (FOXP3) signaling essential for the differentiation and function of T-regs [44]. HIV also downregulated TLR4 and FASLG, which are key in caspase signaling upon interactions with death receptors. In our study, the IL-10 pathway was consistently activated in HBECs upon all treatments (S1, HIV, and combination). This finding is consistent with observed increases in IL-10 expression in HIV-infected young individuals who contracted SARS-CoV-2 [45]. There is evidence that IL-10 regulation of HIV-specific CD4 and CD8 T cell functions [46], which may help explain why decreased SARS-CoV2 replication was associated with increased IL-10 in co-infection assays performed in vitro by Vanetti et al. [45]. Our results are also consistent with those observations, as there was no indication of increased risk of COVID-19 illness in HIV young individuals [45].

Notably, this study is limited by the use of recombinant spike protein from the SARS-CoV-2 Alpha variant. With the constant emergence of SARS-CoV-2 variants, timely sequencing of variants becomes as crucial for surveillance as for learning about the impact of those in host cells. In the case of HIV-SARS-CoV-2 co-infections, studying the implications of these two pathogens together present two moving targets, given the continuous evolution of both viruses. Nevertheless, our studies contribute to the field by demonstrating that experiments with recombinant spike protein invoke cytokine storms similar to that seen in COVID-19, and that appropriate cell culture conditions must be considered according to the physiological context of the cells used in culture. Moreover, we compare, for the first time, the cytokine storms invoked by spike, HIV, or combinations in HBECs to describe the nature of the first-line mucosal responses commanded by the respiratory epithelium in PLWH.

We used gene expression as experimental readouts; however, changes in gene expression in cells treated with spike protein must not be taken out of context. Our results do not suggest that the spike protein, which is the basis of the mRNA vaccines for COVID, enters the nucleus of the host cells to change genes. Our work supports others’ by showing that the spike, as an antigen, changes the way that human cells respond to their microenvironment and in this case, it reinforces antiviral mechanisms as the first line of defense in the airway.

## Figures and Tables

**Table 1:** **Primers used to evaluate ALI differentiation stages**. The primers in this table were used for screening changes in gene expression hallmarks of ALI differentiation.

Official Name	Official Symbol	Reference Position (Accession Number)	GeneGlobe ID	Hallmark
Glyceraldehyde-3-phosphate dehydrogenase	GAPDH	856 (NM_001256799)	PPH00150F-200	Housekeeping
Ribosomal protein, large, P0	RPLP0	921 (NM_001002)	PPH21138F-200	Housekeeping
Angiotensin I converting enzyme 2	ACE2	2449 (NM_021804)	PPH02572A-200	SARS-CoV-2 Receptor
Forkhead box J1	FOXJ1	767 (NM_001454)	PPH02286B-200	Cilia
Mucin 5AC, oligomeric mucus/gel-forming	MUC5AC	3775 (NM_001304359)	PPH60210J-200	Goblet Cells
Claudin domain containing 1	CLDND1	692 (NM_001040181)	PPH00517A-200	Tight Junctions
Secretoglobin, family 1A, member 1	SCGB1A1	198 (NM_003357)	PPH02860F-200	Club Cells
Achaete-scute family bHLH transcription factor 1	ASCL1	1135 (NM_004316)	PPH07090B-200	Neuroendocrine Cells

**Table 2:** **Sources of Antibodies and Description of Working Conditions.** This table provides details for all the antibodies used for Western blot in this report including manufacturer, catalog number and mass of the protein on Western blot.

Molecular Target	Molecular Weight (kDa)	Host	Manufacturer	Catalog Number	Working Conditions (Dilution, Temperature, and Time)
MX1	76	Rabbit	Cell Signaling (Danvers, MA, USA)	37849S	1:1000; 4 °C, 16 h
IL-1-alpha	31	Rabbit	Cell Signaling (Danvers, MA, USA)	84618S	1:1000; 4 °C, 16 h
CD14	50	Rabbit	Cell Signaling (Danvers, MA, USA)	56082S	1:1000; 4 °C, 16 h
ACE 2	75,100,150	Rabbit	Abcam (Waltham, MA, USA)	ab15348	1:1000; 4 °C, 16 h
Vinculin	124	Mouse	Santa Cruz Biotech (Dallas, TX, USA)	sc25336	1:10,000; 4 °C, 16 h
Goat anti Rabbit HRP		Goat	ThermoFisher (Rockford, IL, USA)	32460	1:5000; 4 °C, 2 h
Goat anti Mouse HRP		Goat	ThermoFisher (Rockford, IL, USA)	32430	1:5000; 4 °C, 2 h

**Table 3:** **A Summary of Stress and Toxicity Pathway Results**. The top ten genes from the Qiagen RT2 Profiler Stress and Toxicity Pathways array which had fold regulation changes of greater than 1.5 and a significant *p*-value for the 50 ng/mL or 5 μg/mL S1 treatments. These ten genes are out of the 84 genes screened in the RT2 Profiler PCR Array for Human Stress and Toxicity Pathway Finder.

Category	Gene	Description	50 ng/mL S1		5 μg/mL S1	
			Fold Regulation	*p*-Value	Fold Regulation	*p*-Value
Inflammatory Response	CCL2	Chemokine, binds to CCR2 receptor, activates monocyte, lymphocytes, promotes monocyte recruitment	−2.37	0.0489	−2.68	0.082551
	IL-1-A	Proinflammatory cytokine, B-cell maturation/proliferation and fibroblast activity	2.94	0.011538	2.45	0.154436
	IL-1-B	Proinflammatory cytokine, Neutrophil, T-cell and B-cell activation, antibody production, fibroblast proliferation and collagen production	2.62	0.004414	1.99	0.069231
Hypoxia Signaling	MMP9	type IV collagenase, degrades collagen elastin, involved in autoimmune disease	2.02	0.122415	2.07	0.023545
	SERPINE1	inhibitor of fibrinolysis, component of innate antiviral immunity	1.95	0.029093	1.7	0.090944
Osmotic Stress	CFTR	Cystic fibrosis transmembrane conductance regulator, chloride channels in epithelial cells	−1.65	0.071276	−1.98	0.059924
	SLC5A3	Sodium/myo-inositol cotransporter	−1.56	0.059692	−1.41	0.193088
Oxidative Stress	FTH1	Encodes the heavy subunit of ferritin, storage of iron in soluble nontoxic form	1.51	0.07497	1.7	0.007262
Unfolded Protein Response	DDIT3	DNA damage inducible transcript 3, negative inhibitor preventing DNA binding	1.52	0.022741	1.73	0.057586
Other DNA Damage Response	GADD445A	Growth arrest and DNA-damage-inducible	1.67	0.06617	1.79	0.049673

**Table 4:** **Summary of Human and Adaptive Immunity Response pathway results.** HBE cells under ALI conditions for 21 days were treated with 50 ng/mL of recombinant SARS-CoV-2 Spike protein for 4 h. Cells were harvested and processed for RNA extractions and PCR arrays in six-plicates. Fold changes were calculated using the 2^–∆∆Ct^ formula, using a C_T_ cutoff set to 35. Data normalization was done using the geometric means of all three housekeeping genes beta2-microglobulin (B2M), glyceraldehyde-3-phosphate dehydrogenase (GAPDH), and Ribosomal protein lateral stalk subunit P0 (RPLP0). The table shows the top ten genes with statistically significant fold regulation greater than, 2.5.

Gene	Description	50 ng/mL S1	
		Fold Regulation	*p*-Value
MX1	Dynamin-like GTPase, antiviral activity Pro-inflammatory cytokine, cell growth	4.39	0.015358
CXCL10	Regulation, apoptosis and angiostatins defense response to viruses	4.25	0.03704
CCL5	Inflammatory cytokine. Monocyte, T-helper cell, and eosinophil chemoattractant	2.66	0.038203
IL-1β	Pro-Inflammatory cytokine, B-cell maturation/proliferation and fibroblast activity	2.53	0.05476
IL-1α	Pro-inflammatory cytokine, neutrophil, T-cell and B-cell activation, antibody production, fibroblast proliferation and collagen production	2.17	0.052396
DDX58	Pattern-recognition receptors, senses cytoplasmic viral nucleic acids	2.03	0.037154
HLA-A	MHC1 heavy chain, antigen presenting	1.84	0.027322
STAT1	Signal transducer and transcription activator	1.76	0.037244
CD14	Mediates innate response to bacterial LPS	1.63	0.024603

## Data Availability

The data supporting reported results are available upon request to the corresponding authors.
